# Clinicopathological Significance of Heat Shock Protein (HSP) 27 Expression in Gastric Cancer: A Updated Meta-Analysis

**DOI:** 10.1155/2020/7018562

**Published:** 2020-07-22

**Authors:** Tong Liu, Duo Liu, Xiangxue Kong, Mei Dong

**Affiliations:** Harbin Medical University Cancer Hospital, Harbin, China

## Abstract

**Aim:**

HSP27 is a protein chaperone protecting cell from heat shock, and upregulated HSP27 expression has been found in many different cancers. We conduct this update meta-analysis to evaluate the relationship between HSP27 expression and clinicopathological features.

**Methods:**

We searched PubMed, Chinese CNKI, and WanFang databases to identify studies that assessed the association between clinicopathological feature and HSP27 expression in gastric cancer patients.

**Results:**

We found overexpression of HSP27 was associated with incidence of gastric cancer (OR = 6.31, 95% CI = 1.10–36.15, *P* < 0.0001). However, there was no significant difference between HSP27 expression and gastric cancer differentiation, gender difference, lymph node metastasis, and distant metastasis.

**Conclusion:**

Our meta-analysis study indicates that overexpression of HSP27 is associated with incidence of gastric cancer statistically.

## 1. Introduction

Gastric cancer is the second leading cause of cancer-related mortality and the fourth most common cancer globally [1]. In 2014, 410,400 new stomach cancer cases and 293,800 cancer-associated deaths were estimated to have occurred in China. The crude incidence rate of stomach cancer was 30.00/100,000, and the crude mortality rate of stomach cancer was 21.48/100,000 [2]. As many factors impact gastric cancer prognosis, identifying important biomarkers of gastric cancer will have a great influence for patients.

Small heat shock protein (sHSP) is a group of proteins, which express ubiquitously from prokaryotes to eukaryotes. HSP27 exists as a multimeric complex in the cells and serves functions, like refolding of unfolded proteins, regulation of cytoskeleton dynamics, and cell cycle regulation. sHSPs bind to a wide range of cellular proteins and are implicated in several cellular functions, apart from providing protection against various environmental and physical stressors, such as high temperature and chemical toxins [3].

High expression levels of HSP have been reported in many cancers, including breast, head and neck, gallbladder, colorectal, skin, liver, colon, renal, prostate, and ovarian cancer [4, 5]. Of particular interest, HSPs play dual complex role in apoptosis via promoting or counteracting cell death. For instance, HSPs have been shown to activate apoptotic mediators such as procaspase 3 [6, 7], and conversely, they bind and inhibit several molecules at different levels in the apoptotic pathway [8]. The antiapoptotic events include the blockade of cytochrome C release from the mitochondria by HSP27 besides antagonizing caspase 3 and caspase 9 [9–11]. HSP27 can also suppress other apoptotic death receptor pathways, including TNF*α*, Fas, and TRAIL [12].

HSP27 is reported to be a major target in combating cancer. An increased level of HSP27 is reported in different cancers, including breast cancer, endometrial cancer, and leukemia [3]. Although several studies have reported the relationship between HSP27 and gastric cancer, the conclusions are controversial and the patients included in each study are not enough. Therefore, we conducted meta-analysis of those studies to explore the relationship between HSP27 expression and clinicopathological feature of gastric cancer.

## 2. Methods and Materials

As there was a meta-analysis study published before about relationship between HSP27 expression and clinicopathological feature of gastric cancer, we referred to relevant content about method and material of the study [13].

### 2.1. Identification and Eligibility of Relevant Studies

We searched PubMed, Chinese CNKI, and WanFang databases to identify studies that assessed the association between clinicopathological feature and HSP27 expression in gastric cancer patients. The search ended in September 1, 2019. Search words were “heat shock protein 27,” “HSP27,” “gastric cancer,” “gastric carcinoma,” and “stomach neoplasm.”

The included criteria for this study were as follows: (1) patients were diagnosed as gastric cancer; (2) HSP27 expression was tested in tissue of gastric patients by immunohistochemistry (IHC); (3) study design was case-control study or cross-sectional study; (4) studies included at least one primary outcome of interest; and (5) study was published in English or Chinese with full text available.

The excluded criteria for this study were as follows: (1) letters, reviews, conference abstracts, animal experiments, fundamental research, and duplicated studies were excluded; (2) studies that did not estimate the relationship of HSP27 expression and clinicopathological feature were excluded; and (3) studies whose data could not be used for meta-analysis were excluded.

### 2.2. Data Extraction

Two reviewers independently screened all studies to determine the relevant articles meeting the included criteria. Extracted data included the first author's name, publication year, sample size, country, and clinicopathologial features (HSP27 expression, gender, differentiation, lymph node metastasis, and distant metastasis). Disagreements were resolved by the third-party adjudication.

### 2.3. Quality Assessment

The quality of each included case-control study was assessed by the Newcastle–Ottawa scale (NOS), while the quality of cross-sectional study included was assessed by the Agency for Healthcare Research and Quality (AHRQ). Studies with NOS score ≥6 were considered as good quality and with NOS score ＜5 were considered as poor quality. Studies with AHRQ score 8–11 were as good quality, with AHRQ score 4–7 were as moderate quality, and with AHRQ score 0–3 were as poor quality.

### 2.4. Statistical Analysis

Review manager 5.3 software was used to perform the statistical analysis for these meta-analyses. Pooled odds ratios (ORs) with 95% confidence intervals (CIs) were calculated to evaluate the association between HSP27 expression and clinicopathological feature. The heterogeneity was evaluated by the *I*^2^ test. Fixed effects model was used when there was no significant heterogeneity (*I*^2^ ＜ 50%, *P* ≥ 0.1), while the random effect model was chosen if there was significant heterogeneity (*I*^2^ ≥ 50%, *P* < 0,1).

## 3. Results

### 3.1. Eligible Studies

**Figure 1:**
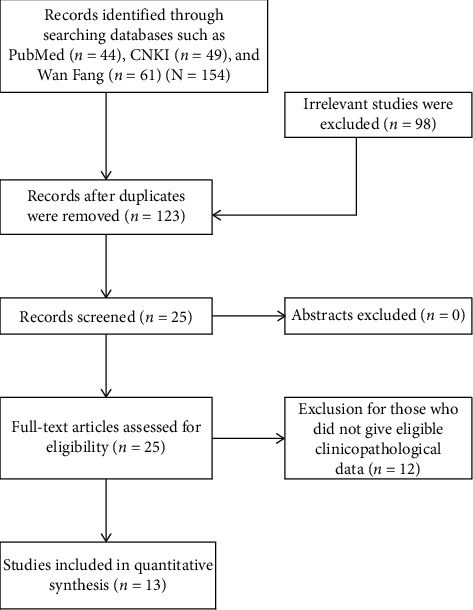
Flowchart of study selection.

As shown in Figure 1, we identified 154 records from PubMed, CNKI, and WanFang databases. After excluding the duplicates and irrelevant studies, 25 studies remained to review the abstracts and full text to find available data. As some data in those studies could not be used, finally, we got 13 studies to conduct meta-analysis to evaluate the relationship between HSP27 expression and clinicopathological feature [14–26].

The characteristics of included studies are shown in Table 1. We got 758 gastric patients, 256 paracancerous tissue specimens, and 230 normal tissue specimens. The included studies were published from 2001 to 2017 and conducted in different countries (ten in China, one in Jordan, one in Japan, and one in Greece). Based on NOS or AHRQ scores, 11 studies were evaluated as good quality, while 2 studies were as moderate quality.

### 3.2. Meta-Analysis

**Figure 2:**
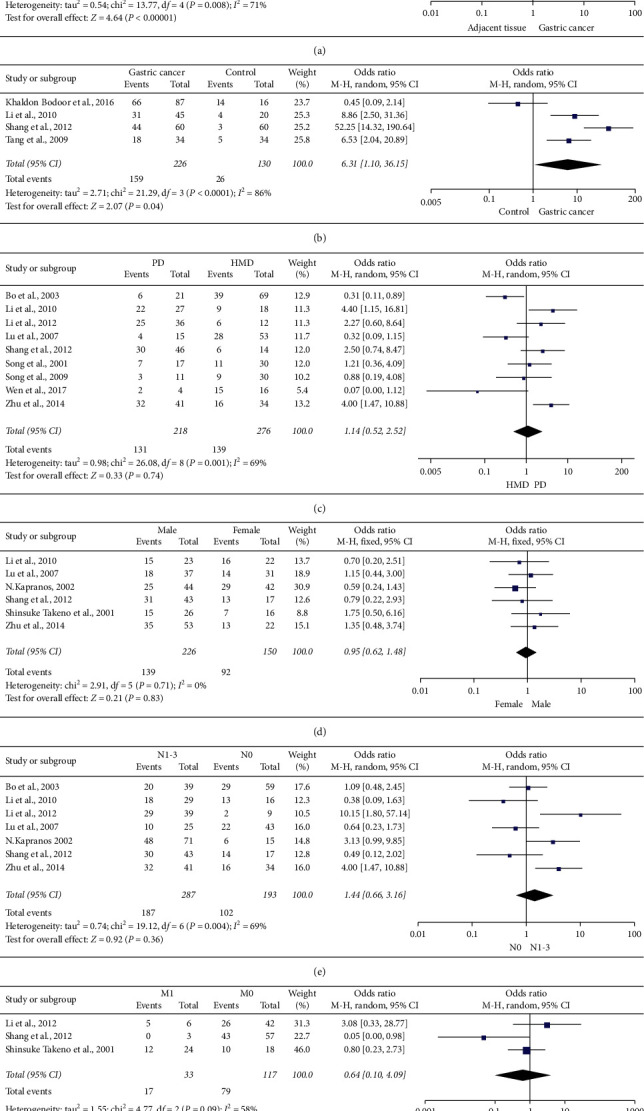
Forest plot of literatures including assessing the relationship between HSP27 expression and clinicopathological features: (a) GC vs AT; (b) GC vs NT; (c) PD vs HMD; (d) male vs female; (e) N1-3 vs N0; (f) M1 vs M0. Abbreviations: GC, gastric cancer; AT, adjacent tissue; NT, normal tissue; PD, poor differentiation; HMD, high or moderate differentiation; random, random effect model; fixed, fixed effect model; OR, odds ratio; and CI, conference interval.

We extracted available data from included studies to conduct meta-analysis. As shown in Table 2 and Figure 2, we assessed the relationship between expression of HSP27 expression and clinicopathological feature of gastric cancer. We found overexpression of HSP27 was associated with incidence of gastric cancer (OR = 6.31, 95% CI = 1.10–36.15, *P* < 0.0001). However, there was no significant difference between HSP27 expression and gastric cancer differentiation (OR = 1.14, 95% CI = 0.52–2.52, *P*=0.74), gender difference (OR = 0.95, 95% CI = 0.62–1.48), lymph node metastasis (OR = 1.44, 95% CI = 0.66–3.16, *P*=0.36), and distant metastasis (OR = 0.64, 95% CI = 0.10–4.09, *P*=0.64).

### 3.3. Publication Bias

**Figure 3:**
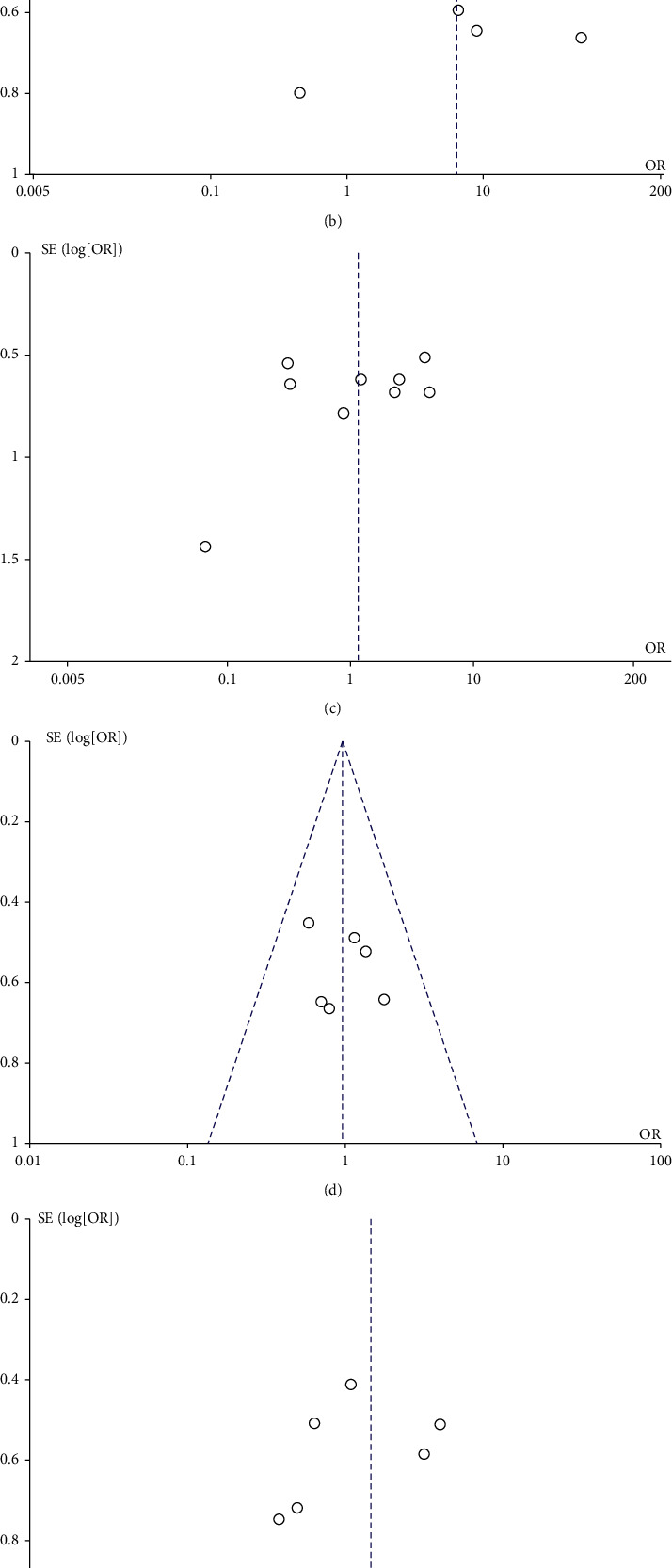
Funnel plot of literatures including assessing the relationship between HSP27 expression and clinicopathological features: (a) GC vs AT; (b) GC vs NT; (c) PD vs HMD; (d) male vs female; (e) N1-3 vs N0; (f) M1 vs M0. Abbreviations: GC, gastric cancer; AT, adjacent tissue; NT, normal tissue; PD, poor differentiation; HMD, high or moderate differentiation; random, random effect model; fixed, fixed effect model; OR, odds ratio; and CI, conference interval.

As shown in Figure 3, we used the funnel plot to assess the publication bias, and we found that there was no significant asymmetry about HSP27 expression in comparison between gastric cancer and normal tissue, male and female, lymph node metastasis and nonlymph node metastasis, and distant metastasis and nondistant metastasis.

## 4. Discussion

We compared our results with previous meta-analysis of the association between HSP27 expression and clinicopathological feature of gastric cancer, which included 9 articles [13]. In our study, we included 13 articles to extract more available data to conduct meta-analysis. Although we added 4 more articles in this meta-analysis based on previous study, we finally got similar results as before. There was statistical significance between overexpression of HSP27 and incidence of gastric cancer. However, we still did not find significance of HSP27 expression in gastric cancer differentiation, genders, lymph node, and distant metastasis.

Previous meta-analysis study combined normal tissue and gastric carcinoma adjacent tissue as the control group. However, considering the difference between normal tissue and gastric carcinoma adjacent tissue, we compared the gastric carcinoma with normal tissue or gastric carcinoma adjacent tissue, respectively. To our surprise, we found there was publication bias in comparison between gastric cancer and its adjacent tissue. The reason might be the different way of choosing and punching biopsy.

As a protein chaperone, HSP27 had many functions in cell, such as antiapoptosis and protecting cell. There were several reports indicating that HSP27 was upregulated in many cancers. Some studies even suggested HSP27 was associated with poor prognosis and was drug resistant, as it could protect tumor cell from apoptosis induced by drugs [27]. In summary, HSP27 might play an important role in cancer therapy and become a new target for treatment in the future.

## 5. Conclusion

Our meta-analysis study indicates that overexpression of HSP27 is associated with incidence of gastric cancer statistically. However, more high-quality research studies with a large sample size should be conducted in future.

## Figures and Tables

**Table 1:** Characteristics of studies included in this meta-analysis.

Author	Year	Country	Study design	No. of samples			Age	Gender: male (±) female (±)	HSP27 expression: gastric cancer (±) adjacent tissue (±) control (±)	Differentiation: low (±) high and middle (±)	Lymph node metastasis: metastasis (±) nonmetastasis (±)	Distant metastasis: metastasis (±) nonmetastasis (±)	Quality score
				Gastric cancer	Adjacent tissue	Normal tissue							
Li et al.	2010	China	ROS	45	30	20	29–70	15/8 16/6	31/14 8/22	9/18 22/27	18/11 13/3	NA	8
Tang et al.	2009	China	ROS	34	34	34	32–84	NA	18/16 4/30	NA	NA	NA	6
Song et al.	2009	China	ROS	41	NA	NA	60–77	NA	NA	3/8 9/21	NA	NA	6
Li et al.	2012	China	ROS	48	NA	14	30–75	NA	31/17	25/11 6/6	29/10 2/7	5/1 26/16	8
Hu et al.	2015	China	ROS	75	75	NA	23–78	35/18 13/9	43/32 62/13	32/9 16/18	32/9 16/18	NA	7
Wen et al.	2017	China	ROS	20	NA	NA	22–86	NA	NA	2/2 15/1	NA	NA	7
Song et al.	2001	China	ROS	54	NA	NA	20–83	NA	NA	7/10 11/19	NA	NA	7
Bo et al.	2003	China	CSS	98	NA	NA	36–78	NA	49/49	6/15 39/30	20/19 29/30	NA	5
Lu et al.	2007	China	ROS	68	57	NA	35–76	18/19 14/17	32/36 15/42 44/16	4/11 28/25	10/15 22/21	NA	7
Shang et al.	2012	China	ROS	60	60	60	33–81	31/12 13/4	6/54 3/57	30/16 6/8	30/13 14/3	0/3 43/14	7
Khaldon Bodoor et al.	2016	Jordan	ROS	87	NA	16	25–98	NA	66/21 14/2	NA	NA	NA	8
Shinsuke Takeno et al.	2001	Japan	CSS	42	NA	NA	42–85	15/11 7/9	NA	NA	NA	NA	6
Kapranos	2002	Greece	ROS	86	NA	86	37–85	25/19 29/13	NA	NA	48/23 6/9	NA	7

**Table 2:** The odds ratio between HSP27 expression and clinicopathological feature of gastric cancer.

Clinicopathological features		Heterogeneity						
		No. of studies	No. of patients	Pooled OR (95% CI)	*P*_Het_	*I* ^2^ (%)	*P* value	Model used
HSP27 expression	GC vs AT	5	173	6.25 (2.88, 13.57)	0.008	71	<0.00001	Random
	GC vs NT	4	159	6.31 (1.10, 36.15)	<0.0001	86	0.04	Random
Differentiation	PD vs HMD	9	270	1.14 (0.52, 2.52)	0.001	69	0.74	Random
Gender	Male vs female	6	231	0.95 (0.62, 1.48)	0.71	0	0.83	Fixed
Lymph node metastasis	N1-3 vs N0	7	289	1.44 (0.66, 3.16)	0.004	69	0.36	Random
Distant metastasis	M1 vs M0	3	96	0.64 (0.10, 4.09)	0.09	58	0.64	Random

Abbreviations: GC, gastric cancer; AT, adjacent tissue; NT, normal tissue; PD, poor differentiation; HMD, high or moderate differentiation; random, random effect model; fixed, fixed effect model; OR, odds ratio; CI, conference interval.
